# Next generation agricultural system data, models and knowledge products: Introduction

**DOI:** 10.1016/j.agsy.2016.09.003

**Published:** 2017-07

**Authors:** John M. Antle, James W. Jones, Cynthia E. Rosenzweig

**Affiliations:** a Oregon State University, USA; b University of Florida, USA; c NASA/Columbia University, USA

**Keywords:** Agricultural systems, Data, Models, Knowledge products, Next generation

## Abstract

Agricultural system models have become important tools to provide predictive and assessment capability to a growing array of decision-makers in the private and public sectors. Despite ongoing research and model improvements, many of the agricultural models today are direct descendants of research investments initially made 30–40 years ago, and many of the major advances in data, information and communication technology (ICT) of the past decade have not been fully exploited. The purpose of this Special Issue of Agricultural Systems is to lay the foundation for the next generation of agricultural systems data, models and knowledge products. The Special Issue is based on a “NextGen” study led by the Agricultural Model Intercomparison and Improvement Project (AgMIP) with support from the Bill and Melinda Gates Foundation.

## Highlights

• This Special Issue lays the foundation for next generation agricultural systems data, models and knowledge products. • Papers are based on a NexGen project led by the Agricultural Model Intercomparison and Improvement Project (AgMIP).
• A vision for NextGen data and models as the basis for a computational agricultural science is presented.
• Foundational papers review the history and state of agricultural science, model design and improvement, and the role of information and computer technologies.
• Topical papers address pest and disease modeling, the models for farm decision support and policy analysis, and new methods to parameterize models.
• The Special Issue concludes with a synthesis and roadmap for implementation of the NextGen vision.

## 1 Introduction

Agricultural system models have become important tools to provide predictive and assessment capability to a growing array of decision-makers in the private and public sectors. Despite ongoing research and model improvements, many of the agricultural models today are direct descendants of research investments initially made 30–40 years ago, and many of the major advances in data, information and communication technology (ICT) of the past decade have not been fully exploited. This state of science is explained in part by the inevitable lag between invention of new ICT tools and their application, but also by an underinvestment in agricultural research, particularly in non-proprietary public good research, and in research aiming to improve the well-being of poor, smallholder farm households in the developing world. At the same time, the private sector continues to utilize ICT developments – such as the recent advances in site-specific management and in the use of “big data” – to improve productivity in large-scale commercial agriculture. Even in commercial applications, advances in data are rapidly exceeding analytical capability. Moreover, these proprietary developments are not contributing little to the publicly available data, models or ICT tools for agricultural systems analysis.

These trends are resulting in a large and growing gap between the potential uses of agricultural system models, particularly in the developing world, and their actual use. This gap between actual and potential model developments and uses presents an opportunity to invest in a new generation of agricultural systems models that could dramatically improve the quality of information available to agricultural decision-makers on the farm, as well as for those making private and public investment and policy decisions.

The purpose of this Special Issue of *Agricultural Systems* is to lay the foundation for the next generation of agricultural systems models and knowledge products. The Special Issue is based on a “NextGen” study led by the Agricultural Model Intercomparison and Improvement Project (AgMIP) with support from the Bill and Melinda Gates Foundation. One of the distinguishing features of the NextGen study methodology was to move from the conventional “supply-driven” approach to model development in which scientists develop models as research tools – to a “demand-driven” or “user-driven” approach that bases data and model developments on the information and decision making needs of the user community. To operationalize this demand-driven approach, the NextGen study began by constructing a set of Use Cases for the development of papers by the study team. These Use Cases were discussed and supplemented by participants at a NextGen stakeholder workshop, where the study papers were presented and discussed. The first of these papers reviewed the state of agricultural systems science and its implications for NextGen models and knowledge products, while the second and third papers discussed various aspects of model and information technology advances.

A second distinguishing feature of the NextGen study was to recognize the need for user-friendly knowledge products, i.e., tools that facilitate the use of model outputs. Knowledge products could take the form of cloud-based analytical tools and mobile technology “apps” or other user-defined forms that would enable the use of the models by a much more diverse set of stakeholders than is now possible.

A third distinguishing feature of NextGen is to devise a proactive strategy to move the agricultural systems modeling community's research agenda in a direction consistent with the NextGen vision. The final paper in this Special Issue synthesizes insights from the articles in this issue and then proposes a NextGen roadmap.

## 2 The NextGen vision: accelerating innovation with computational agricultural science

Our vision is for a new generation of agricultural systems models and knowledge products that can help accelerate the rate of agricultural innovation and meet the global need for food and fiber. Given the stresses now being placed on the air, land, water and genetic resources on which human life depends, these innovations also must help reduce environmental impacts and enhance the resilience of food systems under changing climate conditions. We foresee the use of the new generation of models leading to “virtual” and “computational” agricultural research and development that can complement, and substitute to some degree, for conventional on-the-ground methods. Likewise, significantly improved data and models can contribute to development of advanced farm-management systems, and by making better information available about new systems, could accelerate the adoption and efficient use of more productive and more sustainable technologies. Such data and models are also essential tools for assessing the landscape scale impacts of technologies, evaluating policies to improve resource management, and projecting the performance of technologies under changing climatic and other environmental conditions.

This vision for second-generation models is consistent with research on the past and likely future sources of productivity growth and increases in food commodity production. Research shows that since the agricultural revolution of the mid-20th century, the rate of productivity growth in agriculture has averaged about 2% per year, but this average masks large differences between the high and low-income countries, with productivity levels and growth particularly low in Africa. There is also evidence suggesting that cereal yield growth has been declining over the past several decades. To achieve the goal of sustainable agricultural growth, productivity increases will need to be based on more productive use of genetic resources, land, water, labor and other inputs, not from increasing the amount of land in agriculture or from increasing the use of fossil fuel-based inputs.

The current method for developing innovations in crops, livestock, and agricultural management is based almost entirely on conventional, time- and labor-intensive experimental methods in which new varieties and management practices are evaluated using field-scale experiments that may last for years. On-farm management decisions still depend largely on individual farmer knowledge acquired through personal experience, supported in some cases by “expert” or more formalized decision support. These processes are slow to improve, even with advances in genetic techniques and information technology, and become less effective in environments of increasing uncertainty due to climatic and other changes. Taken together, these conditions suggest that, with appropriate investments, it may be possible to use simulation experiments carried out with models to greatly reduce the need for trial-and-error learning, focusing field experiment to develop capabilities and increase the robustness of simulation tools. These advances could increase the rate of agricultural innovation, and also increase the rate at which these innovations are successfully adopted and implemented on farms.

## 3 Connecting people and models: use cases and knowledge products

There was a consensus among the NextGen study team as well as the participants of the NextGen stakeholder workshop that the next generation of agricultural systems models must be driven by the information needs of diverse stakeholders. To address this challenge, the NextGen study team developed a set of Use Cases to guide their work. The stakeholder workshop validated the authors' Use Cases and developed additional ones that reflect the diverse array of potential users of knowledge products supported by agricultural systems models. The five Use Cases proposed by the NextGen study team guided the critical assessment of the current state of agricultural systems models and the team's thinking about new developments in models and knowledge products.

The Use Cases were created to represent the array of likely users of knowledge products that are linked to NextGen models and data. The five use cases represent two types of farming systems:

### 3.1 Small-holder farms

Small-scale semi-subsistence farms typical of much of Africa and South Asia and other developing regions, many of which produce a mix of subsistence crops, cash crops, livestock, and, in some areas, aquaculture.

### 3.2 Commercial crop enterprises

Large-scale commercially-oriented crop farms typical of the industrialized countries including the United States.

The Use Cases are designed according to the four criteria indicated in Table 1. Narratives further defining the Use Cases are presented in the Appendix A.

**Table 1:** Characteristics of five use-cases.

	Use cases				
	1	2	3	4	5
	Farm extension in Africa	Developing and evaluating technologies for sustainable intensification	Investing in agricultural development projects that support sustainable intensification	Management support for precision agriculture	Supplying food products that meet corporate sustainability goals
Farming system	Small-holder	Small-holder	Small-holder	Commercial crop	Commercial crop
Information user	Farm adviser	Agricultural research team/program	Analyst/adviser	Management consultant	Corporate analyst
Beneficiaries	Farm family	Research institution/farm population	NGO & clients	Farm business	Agri-business firm
Outcomes	Improved livelihood (income, nutrition, food security)	Improved technology	Sustainable technology	Income, soil conservation & water quality	Profit, risk management, sustainability objectives

Table 2 summarizes a number of agricultural system model features that are suggested by the Use Cases. These have important implications for the design of models and knowledge products.

• All of the small-holder use cases (1–3) require whole-farm models, and decision-makers in the commercial crop use cases (4 and 5) are likely to want whole-farm information as well, even if the specific use case (e.g., precision nitrogen application) does not require it.
• All cases need spatially referenced data, but the spatial scale of the data needed varies by case, and all cases need season-specific data. Some farm-level users will need within season data (e.g., for pest management or precision nutrient application).
• All of the Use Cases need biophysical production outputs and economic outputs. The need for environmental and social outputs is case-specific.
• Most, if not all, of the personas in the Use Cases would want to access model outputs via a dashboard application that would probably run on a laptop or larger tablet to facilitate visualization and integration of outputs with other applications and data, although some farm decision-makers or farm advisers might only want mobile applications.
• Only one of the Use Cases might want direct access to model output (the scientist Use Case 2).

**Table 2:** Agricultural system model features indicated by the use cases.

	Use cases				
	1. Farm extension	2. Improved systems	3. Investment in sustainable intensification	4. Precision Ag	5. Sustainable value chains
System features					
- Single production activity	?	?	?	x	x
- Multiple production activities	x	x	x	x	
- Interacting activities	?	?		?	
- Whole farm	x	x	x	?	?
Data (spatially referenced)					
- Single activity	?			x	
- Individual farm	x			x	
- Representative sample		x	x		x
Outputs					
- Bio-physical production (yield)	x	x	x	x	x
- Economic (profit, income)	x	x	x	x	x
- Environmental	?	x	x	?	x
- Social	x	x	x		?
Output access					
- Model		?			
-Mobile app	x			x	
- Computer dashboard	?	x	x	?	x
Spatial scale					
- Field	x	?	?	x	?
- Farm	x	x	x	x	?
- Region (many farms)		x	x		x
Temporal scale					
- Within-season	x	?		x	
- Season	x	x	x	x	x
- Multiple seasons	x		x		x

In our view, this last point is a key revelation for modelers: only one of the personas (the scientist) might want direct access to model output. Moreover, even among the scientists themselves, it is often the case that one user (say, an economist) does not require the output of another model (say, a crop model) in the form it comes out of the model, but would rather have the output put into a format suitable for further manipulation. As these use cases illustrate, this is even more so for non-scientist users: there are few if any users that require direct access to the model output.

As we note below, the paper by Jones et al. (this issue) on *The State of Agricultural Systems Science*, documents that the agricultural models currently available do not meet all of the needs of the five Use Cases. Some provide field-scale predictions of crop responses to management decisions under specified climate conditions. Others provide whole-farm analysis capabilities, but few if any make model outputs accessible through user-friendly means, such as web-based dashboards, that allow users to visualize and interpret model results, compare results across models, or evaluate model uncertainty. The need for this kind of capability was highlighted in the NextGen stakeholder workshop. One of the main messages from current and potential users of model outputs at this workshop was the need to increase relevance and credibility of models as useful tools. Thus, we can conclude that there is a substantial gap to be bridged between current models and the capabilities needed to provide information that would be useful to most potential users. This gap implies the need for a way to link users to models. What we describe as “knowledge products” are the way we envisage that link being made, as discussed further in papers in this Special Issue.

## 4 Overview of the special issue

The first four papers in this Special Issue are foundational papers for the development of NextGen models and are based on the NextGen study team's work and the stakeholder workshop held in August 2014.

The first paper by Jones et al. provides a historical perspective on the development of agricultural systems models, with an emphasis on bio-physically-based models, but also discusses key developments in economic models that related to agricultural system modeling. It also discusses factors that motivated the increases in development of various models and approaches for their applications, highlighting major events and technological advances that led to increases in investments and capabilities of agricultural systems models. It points out how relatively rapid increases in capabilities have occurred in the past, but that capabilities of many agricultural models stagnated for long periods of time, including recent times.

The second paper by Jones et al. provides an overview of the current state of agricultural systems science and modeling. It covers widely-used approaches used for modeling biophysical and economic components and their integration to address broader research questions. It discusses the state of these models and data relative to the needs for knowledge systems to address contemporary issues. Unlike other recent review papers, this one evaluates existing modeling approaches in relation to the needs identified by the NextGen study's use cases presented in this special issue introduction paper. This Jones et al. paper sets the stage for the subsequent papers on approaches needed to produce the next generation of agricultural system models, databases, and knowledge systems for use by a wide range of stakeholder users.

The paper by Antle et al. proposes two main ideas for the development of a new generation of agricultural system models. First is the idea of creating a user-driven approach that would link a more collaborative “pre-competitive space” for model development and improvement to a “competitive space” for knowledge product development. Second, the authors describe some of the potential advances that could be made in the components of NextGen data, models and their integration, as well as possible advances in model evaluation and strategies for model improvement. These two elements form the foundation for the discussion in the final article in this Special Issue which addresses how to move the agricultural modeling community forward towards implementation of these ideas and realization of the NextGen vision.

The paper by Janssen et al. addresses the information and communications technology aspects of next generation models and knowledge products, and describes a framework for development of an open, web-based approach. They argue that improved and new information technology and software can be used by the agricultural modeling community in many ways to address the issues identified in Jones et al. (this issue B) and Antle et al. (this issue). For example, they discuss how it is possible to transition from today's stand-alone, large, and increasingly complex models, to systems models composed of modular model components that could be combined flexibly to represent a wide array of systems. Related software innovations can make possible a collaborative and open approach to model development, reduce costs of model operation and delivery, and broaden access to models and data. They also address the need for better data, through better utilization of existing and new sensor and data collection methods, including remote sensing, crowdsourcing, and mobile technology. They foresee creation of new tools to generate, archive, access, analyze, visualize, and interpret model inputs and outputs. To make these advances possible, new ways of collecting, archiving, and supplying experimental and observational data will be needed to better manage the data side of modeling, and new data standardization, archiving, and access methods that use web-based cloud architectures to simplify and broaden access.

Donatelli et al. address a major gap in existing models, namely the ability to model the effects of pests and diseases on crops. They review the current state of development in coupling pest and disease models to crop models. The authors present a modular approach to disease modeling that illustrates how the modular approach discussed in Antle et al. (this issue) and Janssen et al. (this issue) could be implemented. A roadmap to improve the simulation of the impacts caused by pest and diseases is presented which includes: the quality and availability of data for model inputs and model evaluation; the integration with crop models; methods and processes for model evaluation; and the development of a community of plant pest and disease modelers. The authors conclude with a prospective research project and a set of concrete steps to implement new models.

Capalbo, Antle and Seavert provide an example of the data, models and knowledge products that are being developed to respond to farm-level and policy decision support needs. They describe general features of this type of system and the benefits it could provide to producers as well as research and policy making. They present two models that could be used in this kind of system, a farm-level decision model and a regional policy analysis tool. An application of these models to adaptation of wheat systems in the Pacific Northwest of the United States is used to illustrate the models and the way they could be linked to a “big data” infrastructure. The paper concludes with a discussion of how this type of system could be used to improve the data quality and reduce the cost of data currently collected in the United States and other parts of the world.

Hwang et al. address one of the critical issues identified by Jones et al. (this issue B), the need for better data to support model parameterization. They describe methods for integrating genetic information into crop models through a modular approach. They show that these modules can be developed using mixed effects statistical analyses that quantify the genetic, environment, and genetic by environment interactions that affect plant traits. Ultimately, these modules are integrated together to build new gene-based crop models that will tease out the networks of the genotype by environment by management interactions (G × E × M). An example is described that integrates several gene-based modules to simulate main stem leaf addition on common bean. This research points to the possibility of developing a new generation of crop models that will improve plant breeding, selection of genotypes for specific environments, or alternatively, optimize management of a cultivar grown in a particular environment.

The final paper in the issue provides a synthesis of ideas and discusses a roadmap for their implementation. The goal is to further develop a shared NextGen vision and a proactive strategy to move the agricultural systems research communities and knowledge product user communities towards achieving that vision.

## Appendix A Use case narratives

### 1 Farm extension in Africa

Sizani is working as a farm extension officer in an area in Southern Africa where many farms are very small, incomes are very low, and farmers typically grow maize and beans as staple crops for their family's subsistence and to sell for cash. Some households may have livestock and/or grow vegetables. The aim of the extension service is to help farmers achieve higher and more stable yields of maize and also to advise them on improving their nutrition so that they obtain sufficient protein and micronutrients for healthy families. Sizani obtains information on new varieties of maize and beans that are now available to farmers in the area. These new varieties are more drought and heat-tolerant and the bean varieties are more resistant to a common foliar disease. Sizani also has information on how to improve nutrient management of these crops using small doses of inorganic fertilizer along with animal manure and crop residues. He also has information on a new technique developed by the CGIAR to partially harvest rainfall to increase water availability to the field and vegetable crops. Because farms vary in size, labor availability, soils, and other characteristics, Sizani wants to use the NextGen tools to help tailor advice to each farm family that is practical, likely to be adopted, and provide the best outcome in terms of more stable production, higher income, and better nutrition. Sizani obtains information from the farmer to input into his smart phone, which has NextGen On-Farm Information apps, developed for the farming systems of his region, that help him determine combinations of system components that best fit specific-farm situations. He also has extension information sheets written in the local language that describe the components of crop and farming systems that are likely to succeed with the farm family. In turn, data from the individual farms that Sizani works with are returned to the NextGen model platform to allow for continuous improvement of the tools.

### 2 Developing and evaluating improved crop and livestock systems for sustainable intensification

Francoise is a plant breeder/geneticist working on developing a drought- and heat-tolerant hybrid of maize. She would like to be able to evaluate the potential adoption and impact of maize varieties with particular characteristics across the widely varying conditions in Africa. She realizes, however, that maize is only one part of the complex farming systems used by most farmers, which typically involve multiple crops and livestock. She would like to be able to evaluate the potential of new varieties in these complex systems, rather than evaluating maize by itself as had been typically done by most research programs. Moreover, she would like to know whether the new varieties meet the goals being set up for sustainable intensification, such as improving productivity not just in the short term, but taking longer-term impacts on soils, water, and greenhouse gases into account. Working with a team of colleagues at her research institution, she uses the NextGen Technology Adoption and Impact Assessment Tool for this purpose. This tool integrates the genetic characteristics of the maize varieties with soil, weather, economic and social data representing the farm populations where the new varieties could be used. The research team then simulates the potential for adoption and impacts of the new varieties, providing Francoise with guidance for the kinds of genetic modifications that would be most valuable to farmers, and also provide an assessment of the long-term sustainability of the systems.

### 3 Investment in agricultural development to support sustainable intensification

Stanley is an investment manager for a prominent Foundation, and he needs to evaluate a project for small farms in Kenya that will increase the intensity of production by increasing fertilizer use per hectare on cash crops while maintaining the current sustainable nutrient balance between pasture grasses, crop residues and animal manure.

Before authorizing a project that combines extension information and fertilizer subsidies, Stanley wants to evaluate whether the higher crop yields would induce a non-sustainable system once the initial period of fertilizer subsidies and extension was completed. Initially he uses the NextGen data and crop and livestock model components to assess the yield and labor impacts of increased yields. An economic assessment model is used to estimate if the current cropping balance will change under the new fertilizer program and if increased fertilizer costs can be more than compensated by increase in cash crop yields in the long run. A long-term farm-level nutrient balance under increased intensification will show whether the new system is sustainable. Stanley would like to evaluate these results under a range of assumptions, and present these to local decision makers so that they share common expectations and uncertainties. For this he uses the NextGen Project Assessor, which opens as a webpage on his computer, and he sets up a new assessment, enters data supplied with the project proposal, and links this to general data layers available in the tool. The Project Assessor then uses the configuration of NextGen model components (both biophysical and socioeconomic) needed to conduct the specific assessment.

### 4 Management support for precision agriculture in the US for profitability, soil conservation and water quality protection

Greg is a farmer in the US, with a large corn/soybean-based operation and a high level of mechanization fully equipped with auto-tracking system and high-resolution differential GPS. His tractors are equipped with on-the-go sensors for variable applications of seeding, fertilizer, pesticide, and herbicide. Harold is Greg's precision agriculture consultant. Greg receives weekly updates on his smart-phone and tablet from Harold's Precision Agriculture Company about the status of his crops obtained from drone flights and crop model predictions using a combination of observed and forecasted weather. Harold's analysis relies on the NextGen models that are able to deliver strategic and tactical crop management strategy recommendations, process-based variable rate prescriptions for fertilizer/pesticide/herbicide application, and accurate harvest recommendations. The variable-rate prescription map created by Harold's company is cloud-based and is automatically integrated in Greg's tractor's automated system for variable rate application of inputs. This system allows Greg to track all the activities performed in the field and link them to the harvested product.

### 5 Supplying food products that meet corporate sustainability goals

Jennifer is an economic analyst in a corporate sustainability group. This group has embarked on efforts to make sustainability the core of their mission: marketing food while conserving resources. She is assessing the life-cycle of food products to find ways to conserve energy, save water, minimize waste and reduce greenhouse gas emissions in an effort to make these products more sustainable from farm to fork. Using a web service, Jennifer works with her analysis team to access the NextGen Supply System Assessment Tool. This tool uses real-time weather and historical climate conditions to identify strategies that will optimize the amount of fertilizer to be used across many locations by the corporations enter into contracts with farmers with the goal of increasing yield and reducing greenhouse gas emissions. Using remote-monitoring solutions, as an integral part of the Next-Gen model platform, along with advanced cloud services, Jennifer can help the corporation's contract farmers with decisions regarding when to plant, when to irrigate and when and how much fertilizer to apply.

